# Evaluation of the Relationship Between Adenomyosis and Cervical Elastography Parameters

**DOI:** 10.3390/jcm15041375

**Published:** 2026-02-10

**Authors:** Dilara Sarikaya Kurt, Ahmet Kurt, Sümeyya Duran Kaymak, Berna Turhan, İzzet Özgürlük, Hüseyin Levent Keskin, Kadriye Erdoğan

**Affiliations:** 1 Department of Obstetrics and Gynecology, Etlik City Hospital, Ankara 06170, Türkiye; mflkurt@gmail.com (A.K.); iozgurluk@gmail.com (İ.Ö.); hlkeskin@yahoo.com (H.L.K.); opdrkerdogan@gmail.com (K.E.); 2 Department of Radiology, Etlik City Hospital, Ankara 06170, Türkiye; drsumeyya@gmail.com (S.D.K.); brn.turhan@hotmail.com (B.T.); 3 Department of Obstetrics and Gynecology, School of Medicine, Ufuk University, Ankara 06805, Türkiye

**Keywords:** adenomyosis, transvaginal ultrasonography cervical elastography, shear-wave elastography

## Abstract

**Objectives:** We aim to investigate cervical biomechanical alterations associated with adenomyosis using shear-wave elastography (SWE), and to explore the discriminative potential of cervical SWE parameters. **Methods:** In this prospective study, 84 patients with adenomyosis, diagnosed both clinically and by ultrasonography according to the MUSA parameters, and 65 healthy women underwent elastography to the cervix with SWE. Six areas of the cervix were evaluated: anterior and posterior internal os, middle part of the cervix, and external os. **Results:** The adenomyosis group showed a significantly higher cervical length (27.3 ± 5.5 mm vs. 23.8 ± 4.6 mm), as well as greater anterior (11.3 ± 2.4 mm vs. 9.9 ± 1.3 mm) and posterior (11.3 ± 2.2 mm vs. 10.5 ± 1.8 mm) cervical measurements compared with the controls (*p* < 0.001). SWE showed higher stiffness measurements for the anterior and posterior internal os (22.3 ± 5.4 kPa and 22.2 ± 4.9 kPa) compared with the controls (15.5 ± 5.8 kPa and 15.7 ± 5.6 kPa, respectively; *p* < 0.001). Receiver operating characteristic analysis demonstrated high discrimination for these measurements, with area under curve values of 0.804 for the anterior internal os and 0.808 of posterior internal os. Optimal cut-offs were 17.5 kPa (sensitivity 82%, specificity 70%) and 18.5 kPa (sensitivity 81%, specificity 74%). **Conclusions:** Cervical elastography may serve as a non-invasive adjunctive tool for exploring disease-related biomechanical changes and for supporting imaging-based assessment of adenomyosis.

## 1. Introduction

Adenomyosis is a gynecological disorder characterized by the abnormal proliferation of endometrial glands and stroma within the myometrial layer of the uterus [1]. It is commonly observed in women of reproductive age and may present with symptoms such as dysmenorrhea, menorrhagia, and chronic pelvic pain [2]. Although the etiopathogenesis of adenomyosis has not been fully elucidated, hormonal factors, inflammation, and invasion mechanisms are thought to play a role in its development. Diagnosis of adenomyosis is primarily based on imaging modalities such as transvaginal ultrasonography (TVUS) and magnetic resonance imaging (MRI), which provide high diagnostic accuracy and have largely replaced histopathological confirmation in women of reproductive age [3].

Cervical elastography is a non-invasive ultrasonographic technique used to assess the mechanical properties of cervical tissue [4]. This method helps objectively identify tissue differences by measuring the stiffness and elasticity characteristics of the cervix. Recently, research has focused on the role of elastography in assessing cervical changes and tissue biomechanics during pregnancy and labor. It has also gained growing attention as a tool for diagnosing gynecological disorders [4,5]. Since the elastic properties of the cervix may be associated with structural changes in the uterus, investigating its relationship with myometrial pathologies such as adenomyosis is of significant importance.

Changes in cervical tissue properties in patients with adenomyosis may affect the biomechanical structure of the cervix [6]. Although it is known that this disease alters the structural integrity of the uterus, its relationship with cervical elasticity parameters remains unclear. While conventional imaging methods play a crucial role in diagnosing adenomyosis, emerging technologies such as elastography can provide additional insights into the biomechanical effects of the disease. Therefore, evaluating the relationship between cervical elastography and adenomyosis may offer new perspectives in the diagnosis and management of the disease.

The primary objective of this study is to investigate alterations in cervical stiffness among women with adenomyosis using shear-wave elastography (SWE), to evaluate the relationship between adenomyosis and cervical tissue characteristics, and to explore the discriminative potential of these elastographic parameters between adenomyosis and healthy controls.

## 2. Material and Methods

### 2.1. Study Design and Participants

This prospective observational study was conducted over a six-month period between 1 November 2023 and 1 May 2024. The study was carried out in the Department of Obstetrics and Gynecology at Ankara Etlik City Hospital. Ethical approval for the study was obtained from the Clinical Research Ethics Committee of Ankara Etlik City Hospital (Ethics Committee Approval No: AEŞH-EK1-2023-630, on 18 October 2023), and written informed consent was obtained from all participants.

The study included patients diagnosed with adenomyosis based on gynecological examination and ultrasonographic assessment, as well as a control group consisting of healthy individuals without a diagnosis of adenomyosis, as detailed in the inclusion and exclusion criteria section.

### 2.2. Diagnosis of Adenomyosis

The diagnosis of adenomyosis was established using an integrated clinical and transvaginal ultrasound-based approach, in accordance with current clinical practice recommendations. All patients presented with clinical symptoms suggestive of adenomyosis, including dysmenorrhea and/or abnormal uterine bleeding. Transvaginal ultrasound was used as the first-line diagnostic modality, and sonographic assessment was performed using standardized criteria defined by the Morphological Uterus Sonographic Assessment (MUSA) consensus [7,8]. In line with contemporary guidelines, histopathological confirmation was not required in women not undergoing hysterectomy, as transvaginal ultrasound has been shown to provide high diagnostic accuracy for adenomyosis when standardized imaging features are applied [9].

### 2.3. Inclusion and Exclusion Criteria

Inclusion criteria for the study were being between 18 and 55 years of age, having received a diagnosis of adenomyosis through gynecological examination and ultrasonographic evaluation, and meeting the diagnostic criteria for adenomyosis according to the Morphological Uterus Sonographic Assessment (MUSA) guidelines [7,8]. All control participants were systematically screened and were included only if they were asymptomatic and showed no clinical or transvaginal ultrasonographic findings suggestive of adenomyosis according to MUSA criteria. Participants were eligible for inclusion only if they had no history of cervical or pelvic infections, pathology, or surgery; systemic diseases, or chronic medication use. Exclusion criteria included pregnancy, severe maternal obesity that could interfere with measurements, and the presence of adnexal pathology (e.g., endometrioma, tubo-ovarian complex) or extrinsic pelvic masses detected during ultrasonographic evaluation. To avoid potential confounding, women with documented diagnosis or ultrasound findings suggestive of endometriosis were excluded. The exclusion of endometriosis was performed by taking into consideration the medical history, gynecological examination, and findings on the transvaginal ultrasound; magnetic resonance imaging was not performed. Uterine comorbidities such as uterine fibroids or endometrial hyperplasia were not systematically analyzed as separate variables, as women with overt pelvic pathology or extrinsic pelvic masses were excluded at enrollment. Therefore, the study population represents a clinically selected cohort without significant concurrent uterine or pelvic conditions that could substantially interfere with cervical biomechanical assessment.

### 2.4. Data Collection and Measurements

Demographic and clinical information of all participants, including age, body mass index (BMI), obstetric and gynecological history, menstrual pattern, smoking, and alcohol use, were recorded in a standardized patient follow-up form. As part of the ultrasonographic evaluation, characteristic features associated with adenomyosis—such as uterine dimensions, myometrial heterogeneity, subendometrial lines, and nodules—were assessed.

### 2.5. Cervical Tissue Assessment Using Shear-Wave Elastography

Elastography measurements were performed using a General Electric (Boston, MA, USA) Logic^®^ E9 ultrasound device by the same experienced operator. A transvaginal ultrasound probe (e.g., 1–6 MHz, C1-6-D) was used in accordance with the manufacturer’s recommended protocol to directly target the tissue of interest. To minimize the potential effects of hormonal variation on cervical tissue properties, all ultrasonographic and shear-wave elastography measurements were performed during the follicular phase of the menstrual cycle (between days 5 and 10) in premenopausal participants.

The internal cervical os was identified using standardized transvaginal ultrasonographic landmarks, defined as the point of transition between the hyperechoic endocervical canal and the lower uterine segment in the sagittal plane. Measurements were obtained only after clear visualization of cervical boundaries, and regions with overt myometrial distortion were deliberately avoided. All assessments were performed by an experienced radiologist with expertise in gynecologic ultrasonography to minimize anatomical misclassification.

All elastography measurements were obtained via a transvaginal ultrasound probe. After insertion, minimal pressure was applied to preserve the natural elastic properties of the tissue. The probe was held steady during measurements, and stable images were obtained after a brief waiting period at each site. Elasticity values were recorded from six anatomical regions of the cervix: anterior external os, anterior mid cervix, anterior internal os, posterior external os, posterior mid cervix, and posterior internal os.

Biometric and Shear-Wave Elastography (SWE) measurements were obtained at a predefined reference plane, and the results were recorded. Reliable SWE measurements were defined as those based on at least six acquisitions, including minimum, maximum, and median values. Reliability criteria included a Reliability Measurement Index (RMI) ≥ 0.4 and an interquartile range to median ratio (IQR/M) ≤ 30%. For each predefined cervical region, six consecutive shear-wave elastography measurements were obtained, and the mean value was used for statistical analysis. Technical failure was defined as the inability to obtain a homogeneous elastogram within the sampling area. Measurements were considered invalid if the Region of Interest (ROI) did not align with the target area or if prominent artifacts interfered with the ROI.

**Figure 1:**
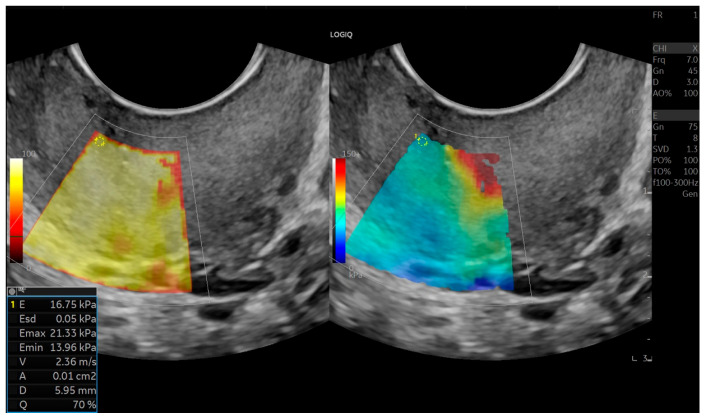
Shear–wave elastography demonstration and measurement of the cervical anterior internal os.

**Figure 2:**
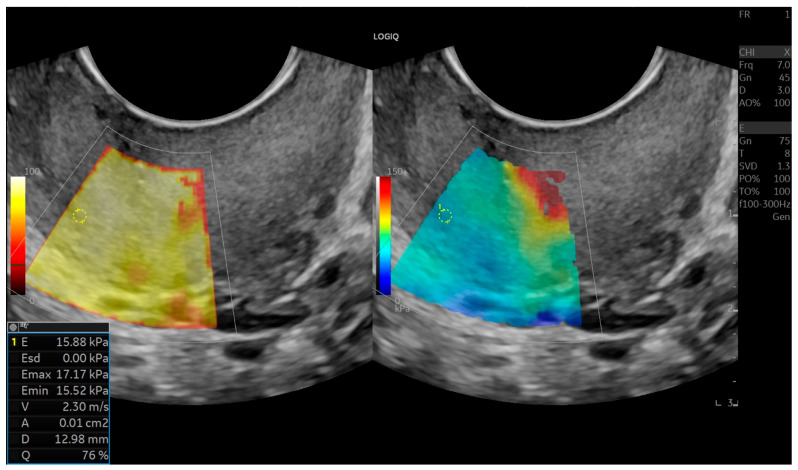
Shear–wave elastography demonstration and measurement of the cervical mid–internal region.

**Figure 3:**
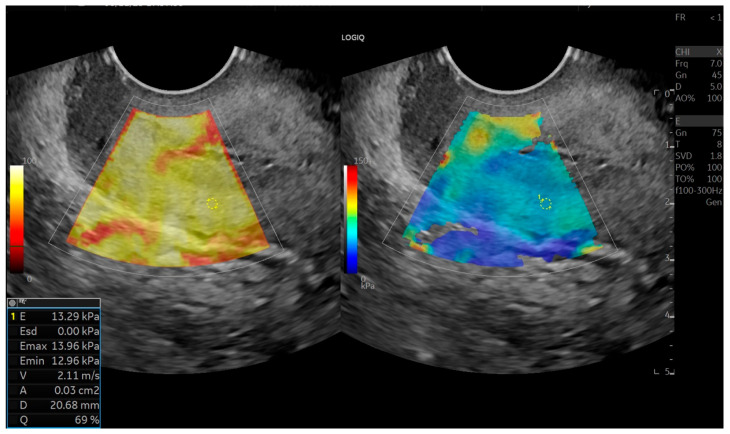
Shear–wave elastography demonstration and measurement of the cervical posterior internal os.

Tissue stiffness in kilopascals (kPa) was calculated based on shear-wave velocity. Cervical elasticity maps were displayed using a color-coded scale: stiffer tissues appeared in red and yellow tones, while softer tissues appeared in blue and green. Elastography data were then compared between patient groups. All measurements were performed by the same experienced radiologist (Figure 1, Figure 2 and Figure 3).

Shear-wave elastography measurement of the internal cervical os obtained via transvaginal ultrasound. The region of interest (ROI) was placed at the anterior internal os, and tissue stiffness was quantified in kilopascals (kPa) based on shear-wave velocity. The color-coded elastography map illustrates tissue stiffness, with warmer colors (red/yellow) indicating stiffer tissue and cooler colors (blue/green) indicating softer tissue.

### 2.6. Sample Size Estimation

As no previous studies specifically evaluating cervical shear-wave elastography parameters in patients with adenomyosis were available at the time of study design, an expected medium effect size was assumed based on methodological recommendations for exploratory clinical studies. Accordingly, an effect size (Cohen’s d) of 0.50 was selected. Sample size estimation was performed using the GPower software (version 3.1.9.7). Using a two-tailed independent samples *t*-test, with an alpha error probability of 0.05 and a desired statistical power of 80%, the minimum required sample size was calculated to be 64 participants per group.

### 2.7. Statistical Analysis

Statistical analyses were conducted using IBM SPSS Statistics for Windows, Version 27.0 (IBM Corp., Armonk, NY, USA). The distribution of continuous variables was assessed using the Kolmogorov–Smirnov test. Variables showing normal distribution were expressed as mean ± standard deviation and compared using the independent samples *t*-test, while non-normally distributed data were analyzed using the Mann–Whitney U test. Categorical variables were presented as frequencies and percentages and compared between groups using the Chi-square test. Elastographic, biometric, and clinical data were examined in both groups using appropriate parametric or non-parametric tests depending on data distribution. Ultrasonographic and elastographic parameters with potential diagnostic value were further evaluated using receiver operating characteristic (ROC) curve analysis, from which optimal cut-off values, sensitivity, specificity, and area under the curve (AUC) values were derived. Variables showing significance in univariate analysis and those considered clinically relevant—specifically, age, body mass index (BMI), and parity—were included as covariates in a multivariable binary logistic regression model, together with cervical biometric and stiffness measurements, to assess their independent association with adenomyosis. Adjusted odds ratios (aORs) and 95% confidence intervals (CIs) were calculated. The level of statistical significance was set at *p*  <  0.05.

## 3. Results

The mean age of patients in the adenomyosis group was 44.8 ± 6.2 years, which was higher than in the control group (41.8 ± 9.2 years; *p* = 0.020). Body mass index was higher in the adenomyosis group (29.2 ± 5.4 kg/m^2^) compared to the control group (27.2 ± 6.0 kg/m^2^) (*p* = 0.031). Obesity was significantly more prevalent in the adenomyosis group compared with controls (44.0% vs. 26.2%, *p* = 0.024). Menstrual irregularity was found in 52.4% of the adenomyosis patients, compared to 24.6% in the control group (*p* < 0.001). Abnormal uterine bleeding was found in 77.4% of adenomyosis patients compared to 35.4% in the control group (*p* < 0.001). Menstrual spotting was found in 57.1% of the adenomyosis group compared to 20% of the control group (*p* < 0.001) (Table 1).

Table 2 summarizes lesion localization, distribution pattern, and extent of adenomyotic involvement based on standardized morphological features defined by the MUSA criteria. In 85.7% of participants, the lesion distribution was evaluated as diffuse, while lesion differentiation was considered diffuse in 84.5% of cases. The presence of cystic components was identified in 71.4% of subjects. Junctional zone involvement was observed in 75%, and mid-myometrial involvement in 84.5% of the cases. Regarding the extent of adenomyosis, 51.2% of cases involved 25–50% of the uterine wall, whereas in 33.3% of cases, more than 50% of the uterine wall was affected.

Table 3 presents the distribution of key ultrasonographic features associated with adenomyosis as described by the MUSA consensus statement. Hyperechoic areas were identified in 89.3% of participants, while fan-shaped acoustic shadowing was detected in 71.4% of cases. Cystic structures were observed in 54.8% of the subjects. A globular uterine appearance was noted in 32.1%, and asymmetric myometrial thickening in 35.7% of cases. The presence of an echogenic subendometrial line was recorded in 33.3% of cases. An irregular junctional zone appearance was identified in 50%, and a disrupted junctional zone was observed in 8.3% of participants. Translesional vascularity was detected in 14.3% of cases (Table 3).

In the adenomyosis group, uterine volume (179.8 ± 111.6 cm^3^) was significantly greater compared with the control group (109.9 ± 78.3 cm^3^; *p* < 0.001). Cervical length (27.3 ± 5.5 mm vs. 23.8 ± 4.6 mm; *p* < 0.001), anterior cervical thickness (11.3 ± 2.4 mm vs. 9.9 ± 1.3 mm; *p* < 0.001), and posterior cervical thickness (11.3 ± 2.2 mm vs. 10.5 ± 1.8 mm; *p* = 0.023) were also significantly higher in the adenomyosis group. Regarding elastographic measurements, both anterior internal os (22.3 ± 5.4 kPa vs. 15.5 ± 5.8 kPa) and posterior internal os (22.2 ± 4.9 kPa vs. 15.7 ± 5.6 kPa) stiffness values were significantly elevated in the adenomyosis group (*p* < 0.001 for both regions) (Table 4).

**Figure 4:**
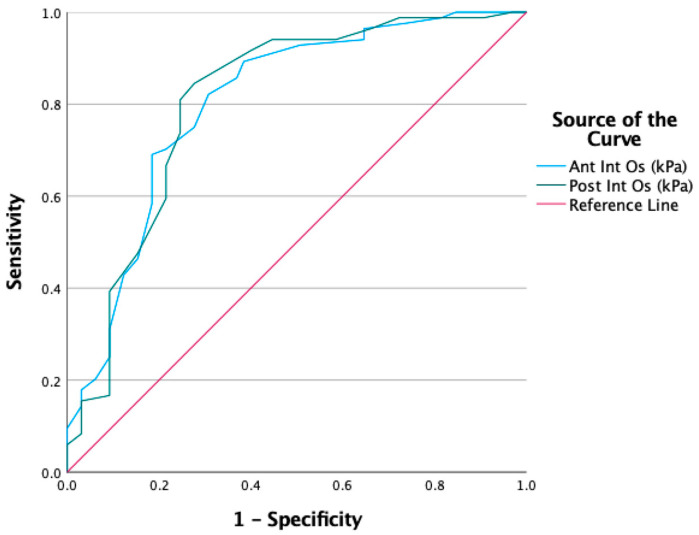
ROC curve showing the discriminative performance of anterior and posterior internal os elastography values in the diagnosis of adenomyosis.

Based on the findings, we generated an ROC curve to demonstrate the discriminative performance of elastography measurements obtained from the anterior and posterior internal os regions in identifying adenomyosis. The curves corresponding to the anterior internal os (Ant Int Os) and posterior internal os (Post Int Os) were evaluated based on their area under the curve (AUC) values, which reflect diagnostic accuracy. The results demonstrate that both regions exhibit high sensitivity and specificity, indicating that elastographic parameters in these areas are effective in discriminating between groups with adenomyosis (Figure 4).

For the anterior internal os (Ant Int Os), the area under the curve (AUC) was calculated as 0.804, demonstrating significant identifying adenomyosis at a cut-off value of 17.5 kPa, with 82% sensitivity and 70% specificity (*p* < 0.001; 95% CI: 0.731–0.878). For the posterior internal os (Post Int Os), the AUC was determined to be 0.808, yielding 81% sensitivity and 74% specificity at a cut-off value of 18.5 kPa (*p* < 0.001; 95% CI: 0.733–0.883) (Table 5).

After adjusting for age, parity, and BMI, an anterior internal os stiffness value greater than 17.5 kPa was significantly associated with an increased likelihood of adenomyosis (adjusted OR: 35.36; 95% CI: 9.99–124.97; *p* < 0.001). Cervical length (aOR: 1.273; 95% CI: 1.124–1.443; *p* < 0.001) and anterior cervical thickness (aOR: 1.892; 95% CI: 1.260–2.840; *p* = 0.002) were independently and positively associated with adenomyosis (Table 6).

## 4. Discussion

In the present study, cervical stiffness parameters measured by shear-wave elastography were significantly higher in women with adenomyosis compared with healthy controls. In particular, cervical length, anterior and posterior cervical thickness, as well as tissue stiffness in the anterior and posterior internal os regions, were significantly higher in the adenomyosis group. Among these parameters, the mean and internal os stiffness values demonstrated the strongest discriminative performance, with AUC values exceeding 0.80.

The increased uterine volume, together with extensive myometrial heterogeneity, presence of cystic components, and irregular junctional zone appearance as defined by MUSA criteria, indicates widespread structural remodeling of the uterine tissue in adenomyosis. These findings suggest that the structural changes in the uterus that develop alongside adenomyosis also affect the cervix, meaning that the cervix is not independent of this process. In this context, cervical elastography may serve as a potential marker for evaluating adenomyosis. The quantitative evaluation of cervical stiffness by SWE may provide complementary information to the morphological features described by the MUSA criteria. Incorporating elastographic parameters—particularly internal os stiffness values—may provide complementary biomechanical information in cases with equivocal sonographic findings; however, these findings should be interpreted in the context of adjacent uterine pathology. Nevertheless, further studies systematically comparing MUSA-based diagnostic accuracy with and without elastography are required to establish its clinical utility.

The cervical stroma contains different collagen and smooth muscle fiber distributions that vary across its different areas. The internal cervical os (ICO) surrounding area stands as the most rigid section among these regions [10]. The anatomical structure of the ICO has been reported to be composed of radially arranged collagen fibers and circumferentially arranged collagen and smooth muscle fibers. Moreover, the muscle fibers in this area have been reported to be responsive to neurotransmitters and oxytocin [11,12]. The ICO functions as the main cervical structure, which opposes stretching forces during pregnancy because of its specific structural design. The elastography method enables researchers to measure tissue elasticity through its assessment of tissue deformability. A reduction in stiffness, indicating increased elasticity of the ICO, typically occurs prior to the onset of labor and childbirth.

The disruption of the endometrial–myometrial interface may contribute to the development of adenomyosis, particularly involving the inner layers of the myometrium [13]. A comparable pathophysiological mechanism might underlie the increased severity of menstrual pain [14] and the elevated incidence of adenomyosis [15], observed in women with a more pronounced retroflexion of the uterus, a condition that may also impede menstrual outflow. The hypothesis that increased stiffness of the internal cervical os (ICO) results from adenomyosis appears less plausible. In fact, greater ICO stiffness has been documented in individuals experiencing severe dysmenorrhea even in the absence of ultrasonographic indicators of adenomyosis [10]. Furthermore, adenomyosis has been correlated with a heightened likelihood of caesarean delivery [16]. Therefore, future investigations should aim to clarify the potential association between ICO stiffness and the preferred mode of childbirth.

As previously documented, the prevalence of adenomyosis tends to increase with advancing age [17,18,19,20], and it was observed more frequently among women undergoing gonadal steroid therapy, likely due to the therapeutic management of their clinical symptoms [10]. Coexisting conditions commonly linked with adenomyosis were intentionally excluded from the logistic regression models, as the primary objective was to explore a potential underlying mechanism of pathogenesis. Although some research indicates that adenomyosis and endometriosis might share similar pathogenic pathways and may independently influence uterine or cervical tissue stiffness [21,22], endometriosis was not incorporated into the current analysis for this reason.

Although studies investigating adenomyosis using shear-wave elastography are limited, Acar et al. [23] reported significantly increased myometrial stiffness in women with adenomyosis, demonstrating high diagnostic accuracy (AUC = 0.908). In contrast, our study focused on the cervical compartment and showed that cervical stiffness also increases in adenomyosis. These results suggest that adenomyosis may affect not only the myometrium but also the cervix as part of a broader uterine remodeling process. Therefore, cervical elastography could complement myometrial assessment and improve the noninvasive diagnosis of adenomyosis.

Xholli et al. (2023) reported that cervical stiffness at the internal cervical os (ICO) region was significantly increased in women with adenomyosis, and that this stiffness was independently associated with the presence of adenomyosis [24]. Similarly, in our study, elastographic values in both the anterior and posterior internal os regions were found to be significantly elevated, and these regions demonstrated high discriminative performance. A shared conclusion of both studies is that the ICO region represents a critical anatomical site reflecting the biomechanical effects of adenomyosis. These parallel findings suggest that alterations in cervical tissue stiffness may play a significant role in the pathophysiology of adenomyosis, and that elastographic assessment of this region could offer a valuable contribution to diagnosis. Furthermore, the hypothesis proposed by Xholli and colleagues—that a “stiff ICO may impede menstrual flow, thereby increasing uterine contractility and indirectly contributing to adenomyotic invasion”—provides a pathophysiological explanation that is consistent with the increased cervical stiffness identified in our study. However, unlike their study, which relied solely on qualitative strain assessment, our research employed shear-wave elastography to obtain quantitative stiffness measurements across multiple cervical regions. This approach provided a more objective evaluation of cervical biomechanics. Furthermore, by providing quantitative stiffness measurements across multiple cervical regions, our study adds objective data to the existing literature regarding cervical biomechanics in adenomyosis. However, although elastographic parameters demonstrated good discriminative performance, the present study was not designed to determine whether shear-wave elastography improves diagnostic accuracy beyond established imaging criteria. Therefore, cervical elastography should be considered an adjunctive and exploratory tool rather than a standalone diagnostic modality. In particular, the potential influence of adenomyotic lesions extending toward or abutting the lower uterine segment should be considered when interpreting cervical stiffness measurements.

In the case where histopathological examination is used in diagnosing adenomyosis, it must be understood that there are no set standards in the examination of excised uterine tissue [22]. However, studies that utilized clear and methodologically precise protocols have shown that transvaginal ultrasonography may have accuracy rates that exceed 80%, with specificity rates that may reach 98% [7,8,9,25,26,27]. Future studies must be conducted to compare the diagnostic capability of individual ultrasonography markers, combinations of markers, and detailed histopathological examinations. Most importantly, it must be emphasized that hysterectomy is not suitable in cases where fertility preservation is desired.

Beyond imaging-based diagnostic strategies, recent studies have highlighted the potential role of non-invasive, biomarker-based approaches in adenomyosis. In particular, circulating and biofluid-derived microRNAs have emerged as promising candidates. A recent machine learning-based pilot study demonstrated that distinct serum and urine miRNA signatures could discriminate adenomyosis from endometriosis and negative controls, with urine-derived profiles showing the highest diagnostic performance [28]. In parallel, a comprehensive systematic review has shown that dysregulated non-coding RNAs are involved in key pathogenic processes of adenomyosis, including epithelial–mesenchymal transition, fibrosis, immune modulation, and extracellular matrix remodeling [29]. These molecular alterations may intersect with biomechanical tissue changes, suggesting that elastography and molecular biomarkers capture complementary aspects of the same disease process. While imaging-based assessment using TVUS and MUSA criteria remains the diagnostic cornerstone, biomarker-based approaches are still investigational. Cervical shear-wave elastography may therefore represent a practical, non-invasive adjunct to imaging, and future studies integrating elastographic and molecular data may help establish a more comprehensive diagnostic framework for adenomyosis.

This study has several limitations. First, it was conducted at a single center with a limited sample size, which may restrict the generalizability of the findings. Although elastographic measurements were performed by a single experienced operator, potential inter- and intra-observer variability remains a limitation due to the inherently operator-dependent nature of this technique. In addition, cervical elasticity measurements were performed during the follicular phase for standardization; however, the overall influence of hormonal fluctuations on cervical tissue properties could not be fully evaluated. Moreover, this study focused solely on the elasticity characteristics of the cervix and did not include a detailed analysis of the structural relationship with other uterine segments. Although standardized transvaginal ultrasonographic landmarks were used to define cervical boundaries, in cases of advanced adenomyosis with marked lower uterine segment involvement, partial overlap between cervical tissue and adjacent myometrium cannot be entirely excluded. This potential anatomical ambiguity represents an inherent limitation of ultrasound-based measurements and may have influenced cervical length, thickness, and stiffness assessments. Considering these limitations, future studies with multicenter designs, larger sample sizes, and control of variables such as menstrual cycle phase are warranted. Furthermore, all patients with known or sonographically suspected endometriosis were excluded to minimize etiologic heterogeneity; however, this approach may have limited the generalizability of the findings by excluding endometriosis-associated adenomyosis subtypes, such as those described in Kishi’s classification. Finally, given the limitations of routine ultrasonography in detecting deep infiltrating or posterior compartment endometriosis, the presence of occult endometriosis cannot be entirely excluded, and residual confounding may therefore exist.

## 5. Conclusions

This study demonstrated a significant increase in cervical tissue stiffness in women with adenomyosis, particularly at the anterior and posterior internal cervical os regions. These findings indicate that cervical biomechanical alterations accompany uterine remodeling in adenomyosis and can be quantitatively assessed using shear-wave elastography. Although cervical elastography should be considered an adjunctive and exploratory tool rather than a standalone diagnostic method, it may provide complementary biomechanical information to conventional imaging-based assessment. Further prospective studies including diagnostically challenging cases are warranted to clarify its incremental clinical value.

## Figures and Tables

**Table 1:** Comparison of demographic, clinical, gynecological, and obstetric characteristics between adenomyosis and control groups.

		Control (*n* = 65)	Adenomyosis (*n* = 84)	*p* Value
**Age (years)**		41.8 ± 9.2	44.8 ± 6.2	**0.020 ^a^**
**Gravida**		2.8 ± 1.3	3.0 ± 1.2	0.739 ^b^
**Parity**		2.3 ± 1.0	2.5 ± 1.2	0.868 ^b^
**BMI (kg/m^2^)**		27.2 ± 6.0	29.2 ± 5.4	**0.031 ^a^**
**Obesity**		17 (26.2%)	37 (44.0%)	**0.024**
**Smoking (pack-years)**		5.3 ± 8.2	3.9 ± 7.4	0.325 ^b^
**Menstrual pattern**	Irregular	16 (24.6%)	44 (52.4%)	**<0.001**
	Regular	49 (75.4%)	40 (47.6%)	
**History of uterine surgery**	No	51 (78.5%)	52 (61.9%)	0.064
	CS	14 (21.5%)	30 (35.7%)	
	Myomectomy	0 (0.0%)	2 (2.4%)	
**AUB**	No	42 (64.6%)	19 (22.6%)	**<0.001**
	Yes	23 (35.4%)	65 (77.4%)	
**Intermenstrual spotting**	No	52 (80.0%)	36 (42.9%)	**<0.001**
	Yes	13 (20.0%)	48 (57.1%)	
**Dyspareunia**	No	59 (90.8%)	68 (81.0%)	0.094
	Yes	6 (9.2%)	16 (19.0%)	
**Uterine position**	Antevert	49 (75.4%)	71 (84.5%)	0.162
	Retrovert	16 (24.6%)	13 (15.5%)	

Data are presented as mean ± standard deviation or counts (percentages), where appropriate. CS refers to Cesarean Section and AUB stands for Abnormal Uterine Bleeding. Statistically significant *p*-values are shown in bold. The superscript letters indicate the type of statistical test used: ^a^: Independent samples *t*-test, and ^b^: Mann–Whitney U test.

**Table 2:** Distribution of uterine and lesional morphologic features in patients with adenomyosis.

		Count	Column *N*%
**Lesion localization**	Anterior	8	9.5%
	Posterior	2	2.4%
	Diffuse	72	85.7%
	Focal	2	2.4%
**Lesion differentiation**	Focal	7	8.3%
	Mix	6	7.1%
	Diffuse	71	84.5%
**Presence of cystic component**	No	24	28.6%
	Yes	60	71.4%
**Junctional zone involvement**	No	21	25.0%
	Yes	63	75.0%
**Middle myometrium involvement**	No	13	15.5%
	Yes	71	84.5%
**Outer myometrium (subserosal) involvement**	No	82	97.6%
	Yes	2	2.4%
**Full-thickness uterine involvement**	No	82	97.6%
	Yes	2	2.4%
**Extent of adenomyosis**	<25%	13	15.5%
	25–50%	43	51.2%
	>50%	28	33.3%

Data are presented as counts (percentages). The abbreviation used in the table is as follows: “JZ” stands for Junctional Zone.

**Table 3:** Distribution of ultrasonographic imaging findings in patients with adenomyosis.

		Count	Column *N*%
**Globular uterus appearance**	No	57	67.9%
	Yes	27	32.1%
**Asymmetrical myometrial thickness**	No	54	64.3%
	Yes	30	35.7%
**Presence of cysts**	No	38	45.2%
	Yes	46	54.8%
**Presence of hyperechogenic areas**	No	9	10.7%
	Yes	75	89.3%
**Presence of fan-shaped shadowing**	No	24	28.6%
	Yes	60	71.4%
**Presence of echogenic subendometrial line**	No	56	66.7%
	Yes	28	33.3%
**Presence of translesional vascularity**	No	72	85.7%
	Yes	12	14.3%
**Irregular junctional zone**	No	42	50.0%
	Yes	42	50.0%
**Interrupted junctional zone**	No	77	91.7%
	Yes	7	8.3%

Data are presented as counts (percentages).

**Table 4:** Comparison of ultrasonographic and cervical elastographic parameters between adenomyosis and control groups.

	Control (*n* = 65)	Adenomyosis (*n* = 84)	*p* Value
	Mean ± S.D.	Mean ± S.D.	
**Uterine Volume (cm^3^)**	109.9 ± 78.3	179.8 ± 111.6	**<0.001 ^a^**
**Endometrial thickness (mm)**	7.6 ± 4.2	8.2 ± 4.4	0.475 ^a^
**Cervical Length (mm)**	23.8 ± 4.6	27.3 ± 5.5	**<0.001 ^a^**
**Anterior Cervical Thickness (mm)**	9.9 ± 1.3	11.3 ± 2.4	**<0.001 ^a^**
**Posterior Cervical Thickness (mm)**	10.5 ± 1.8	11.3 ± 2.2	**0.023 ^a^**
**Anterior Cervical Angle (degrees)**	102.9 ± 19.8	109.5 ± 28.9	0.116 ^a^
**Posterior Cervical Angle (degrees)**	135.6 ± 17.9	141.1 ± 26.8	0.160 ^a^
**Utero-Cervical Angle (degrees)**	135.5 ± 31.2	131.0 ± 25.7	0.176 ^b^
**Anterior External Os (kPa)**	14.1 ± 5.2	15.1 ± 4.2	0.201 ^a^
**Anterior Mid-Cervical Region (kPa)**	15.8 ± 5.1	16.5 ± 4.1	0.362 ^a^
**Anterior Internal Os (kPa)**	15.5 ± 5.8	22.3 ± 5.4	**<0.001 ^a^**
**Posterior External Os (kPa)**	14.0 ± 4.9	15.0 ± 4.0	0.158 ^a^
**Posterior Mid-Cervical Region (kPa)**	15.4 ± 4.6	16.4 ± 4.0	0.133 ^a^
**Posterior Internal Os (kPa)**	15.7 ± 5.6	22.2 ± 4.9	**<0.001 ^a^**

Data are presented as mean ± standard deviation. Statistically significant *p*-values are shown in bold. ^a^: Independent samples *t*-test. ^b^: Mann–Whitney U test.

**Table 5:** ROC analysis results of cervical elastography parameters for the discriminative performance in identifying adenomyosis.

	Area	Sensitivity	Specificity	Cut-Off	*p* Value	Asymptotic 95% CI	
						Lower Bound	Upper Bound
**Ant Int Os (kPa)**	**0.804**	**82%**	**70%**	**17.5**	**<0.001**	0.731	0.878
**Post Int Os (kPa)**	0.808	81%	74%	18.5	**<0.001**	0.733	0.883

Data are presented as area under the curve (AUC), sensitivity, specificity, cut-off values, and 95% confidence intervals. The abbreviation “Ant Int Os” refers to Anterior Internal Os, and “Post Int Os” refers to Posterior Internal Os. Statistically significant *p*-values are shown in bold.

**Table 6:** Multivariable logistic regression analysis of factors associated with adenomyosis diagnosis.

	B	*p* Value	Adj. OR	95% C.I.	
				Lower	Upper
**Anterior internal os (>17.5 kPa)**	**3.565**	**<0.001**	35.36	9.99	124.97
**Age (years)**	0.056	0.097	1.058	0.990	1.130
**Parity**	−0.115	0.641	0.891	0.549	1.447
**BMI (kg/m^2^)**	−0.008	0.864	0.992	0.901	1.092
**Cervical length**	0.242	**<0.001**	1.273	1.124	1.443
**Anterior cervical thickness**	0.637	**0.002**	1.892	1.260	2.840
**Posterior cervical thickness**	−0.373	0.055	0.689	0.471	1.008

## Data Availability

Due to hospital policies, patient data and study materials cannot be shared. However, the data are available from the corresponding author upon reasonable request.

## Abbreviations

AUC	Area Under the Curve
BMI	Body Mass Index
CI	Confidence Interval
ICO	Internal Cervical Os
IQR	Interquartile Range
MRI	Magnetic Resonance Imaging
MUSA	Morphological Uterus Sonographic Assessment
OR	Odds Ratio
ROI	Region of Interest
ROC	Receiver Operating Characteristic
RMI	Reliability Measurement Index
SWE	Shear-Wave Elastography
TVUS	Transvaginal Ultrasonography
